# Initial psychological responses to Influenza A, H1N1 ("Swine flu")

**DOI:** 10.1186/1471-2334-9-166

**Published:** 2009-10-06

**Authors:** Robin Goodwin, Shamsul Haque, Felix Neto, Lynn B Myers

**Affiliations:** 1 Social Sciences, Brunel University, Uxbridge UK; 2 School of Medicine and Health Sciences, Monash University Sunway Campus Jalan Lagoon Selatan, Malaysia; 3 Faculdade de Psicologia e de Ciencias da Educacao, Universidade do Porto, Porto, Portugal

## Abstract

**Background:**

The outbreak of the pandemic flu, Influenza A H1N1 (Swine Flu) in early 2009, provided a major challenge to health services around the world. Previous pandemics have led to stockpiling of goods, the victimisation of particular population groups, and the cancellation of travel and the boycotting of particular foods (e.g. pork). We examined initial behavioural and attitudinal responses towards Influenza A, H1N1 ("Swine flu") in the six days following the WHO pandemic alert level 5, and regional differences in these responses.

**Methods:**

328 respondents completed a cross-sectional Internet or paper-based questionnaire study in Malaysia (N = 180) or Europe (N = 148). Measures assessed changes in transport usage, purchase of preparatory goods for a pandemic, perceived risk groups, indicators of anxiety, assessed estimated mortality rates for seasonal flu, effectiveness of seasonal flu vaccination, and changes in pork consumption

**Results:**

26% of the respondents were 'very concerned' about being a flu victim (42% Malaysians, 5% Europeans, p < .001). 36% reported reduced public transport use (48% Malaysia, 22% Europe, p < .001), 39% flight cancellations (56% Malaysia, 17% Europe, p < .001). 8% had purchased preparatory materials (e.g. face masks: 8% Malaysia, 7% Europe), 41% Malaysia (15% Europe) intended to do so (p < .001). 63% of Europeans, 19% of Malaysians had discussed the pandemic with friends (p < .001). Groups seen as at 'high risk' of infection included the immune compromised (mentioned by 87% respondents), pig farmers (70%), elderly (57%), prostitutes/highly sexually active (53%), and the homeless (53%). In data collected only in Europe, 64% greatly underestimated the mortality rates of seasonal flu, 26% believed seasonal flu vaccination gave protection against swine flu. 7% had reduced/stopped eating pork. 3% had purchased anti-viral drugs for use at home, while 32% intended to do so if the pandemic worsened.

**Conclusion:**

Initial responses to Influenza A show large regional differences in anxiety, with Malaysians more anxious and more likely to reduce travel and to buy masks and food. Discussions with family and friends may reinforce existing anxiety levels. Particular groups (homosexuals, prostitutes, the homeless) are perceived as at greater risk, potentially leading to increased prejudice during a pandemic. Europeans underestimated mortality of seasonal flu, and require more information about the protection given by seasonal flu inoculation.

## Background

Medical interest in Influenza A, H1N1 has been considerable [1]. However, despite dramatic warnings in the media, little is known about behavioural responses to pandemic threats. 'Common sense', lay beliefs about those most likely to be at risk, and the appropriate behaviours to adopt to avoid infection, are often not taken into account by medical practitioners. Such beliefs have been shown to influence adherence and self-care behaviours [2]. During the SARS and Ebola outbreaks, association of the viruses with Chinese or African 'others' permitted Europeans to feel relatively safe from infection [3], and contributed to the victimisation of some Chinese in Toronto [4]. Stockpiling by the worried well can rapidly lead to shortages; cancellations of travel and closure of businesses can rapidly have profound economic consequences [5,6]. Faced with concerns about their mortality, individuals may turn to others for reassurance, but these social networks, by sharing their uncertainties, may sometimes contribute to greater stress [7].

The international threat posed by H1N1 calls for a necessary pooling of international data, both by medical teams and by social scientists. Particular concern has been expressed about the pandemic spreading to Asia, and the potential for mixing with other variants, such as avian influenza [8]. Our team in the UK, Portugal and Malaysia sought to explore initial responses to the pandemic influenza threat. Responses to a new pandemic can be very time specific, with public reactions liable to change almost daily with media coverage [9]. Particularly important may be the gathering of data during the escalating responses that accompany a WHO pandemic alert phase 4 and 5 [10]. The WHO raised their flu alert to level 5 on 29^th ^April [11], a mass media information campaign began in the UK on 5^th ^May 2009. We collected data from 30th April 2009 (at which time there had been 9 deaths and 212 confirmed cases worldwide) until 6th May 2009 (31 confirmed deaths, 1569 cases). By 6^th ^May there had been 27 confirmed cases in Europe, but none in Asia.

Our study sought to gather a snapshot of the attitudinal and behavioural responses during the early stages of a pandemic, knowledge about the differences between seasonal and pandemic flu, and those groups seen as most 'at risk' from infection. Understanding such attitudes and levels of knowledge may have important public health implications for information campaigns aimed at encouraging appropriate precautions against infection, while comprehending risk perceptions can help identify those groups most likely to be at risk of stereotyping and prejudice during a pandemic.

## Methods

### Participants and Procedure

Following ethical approval by the relevant University ethics boards in London and Malaysia, data was collected from a total of 328 respondents (mean age 31.2, SD 13.37, 62% female). A paper version of the questionnaire was distributed in Malaysia, with students recruiting 180 respondents from their own classes, and community members from residential areas and local offices in Kuala Lumpur (age range 18-70, mean age 29.0 (SD 13.36), 59% female)). Response rate was generally good, with 180 out of 200 approached to participate (90%) completing the questionnaire. In Europe, data was collected between 30^th ^April and 6^th ^May 2009 from 158 respondents (age range 18-69, mean age 33.9, SD 12.8, 68% female) via an online questionnaire in English or Portuguese, linked to the website http://www.swinefluquestionnaire.com. The website link was pasted onto a variety of general, non-health networking websites (e.g. 'I love London'). Respondents were primarily from the UK and Portugal but also included 30 respondents living outside these countries and resident in Finland (19 respondents), Poland (6 respondents), Malta (3 respondents) and France (2 respondents). Ten non-European based residents were then removed from the online survey before analysis.

### Questionnaire

Respondents were asked to complete a questionnaire 'about perceptions of swine flu' which comprised a number of closed and open-ended format questions. Beliefs about *comparative risk *were analysed for a number of group members associated with risk in previous pandemics. We included these 'high risk' groups on the basis of previous research on representations and reactions to HIV/AIDS, the Ebola virus and SARS [3,9,12-14]. 'Outgroup' members, who may be marginalized by society (prostitutes, homosexuals) have been linked to higher risk in previous epidemics [12,13], while the association of poverty with disease spread in previous infections [3] led us to include homeless people. Concerns about the risk of respiratory diseases from proximity to animals [3] meant we included pig farmers and farmers in general in our list of risk groups. We also included the elderly and immune compromised, two groups at higher risk from seasonal influenzas. Respondents completed closed-format 3-point scales, indicating the extent to which they believed these groups were *more at risk than me, the same risk as me *or *less at risk than me*.

Anxiety indicators were assessed through two closed-format questions assessing personal worries about catching the virus (measured on a 4-point scale, from *very concerned *to *not at all concerned*), as well as questions assessing friends and families' estimates of the risk (4 point scale, from *very high *to *very low*). We used closed format questions for two questions assessing the purchase, or intention to purchase, specific items, such as face masks ("Have you already bought (or are you intending to buy) anything in preparation for a swine flu epidemic (e.g. face masks, food, tissues, cleaning materials)?" (*yes*/*no *responses). We accompanied this with an open-ended question for those who indicated they had/intended to purchase an item ("what have you bought?") with responses categorised for frequency in Malaysia and Europe, with the most common categories reported below. We also assessed whether respondents expected to modify their public transport use as a result of the threat (3 point scale: *increase transport use, decrease transport use*, or *remain the same*, collapsed into the categories 'less' and 'much more or the same' for analysis), and whether respondents intended to cancel or delay travel plans (*yes */*no*). An open-ended question asked 'how can you protect yourself from infection?' with the most frequent responses coded into categories by our research team in Malaysia and Europe.

In Europe only, we asked additional closed-ended questions about stopping or reducing eating pork as a result of the pandemic (*yes*/*no*), and the precautionary desire for anti-viral drugs at home ('would you like to have your own anti-viral drugs at home just in case'? 'Have you tried to obtain any anti-viral medicines to keep just in case?' (both *yes*/*no *answers)). We asked about the mortality rate in ordinary, seasonal flu (five point scale, *under 50,000*, *50-000-99,000*, *100,000-249,000*, *250,000-500,000 *and *over 500,000*), as well as whether the symptoms of swine flu differed from seasonal flu, and whether seasonal flu vaccination provided immunity against swine flu (both *yes*/*no *answers). Throughout, statistical analysis for structured questions was through chi-square analyses and Pearson product moment correlation.

## Results

### Behavioural change

As shown in table 1 approximately a third of respondents reported they would use public transport less (116/320) or had contemplated cancelling or delaying flights (124/312), with this response more pronounced in Malaysia (respective *x*^2 ^(1) = 21.91, 49.20, both *p *< .001). Few had already bought products (25/328), but 41% (74/180) of our Malaysians were preparing to do so (*x*^2 ^(1) = p < .001 for differences between Malaysia and Europe). Most likely to be purchased were masks (mentioned 46 times in Malaysia, 14 in Europe) and food (14 times Malaysia, 5 times in Europe). Asked in the free response question how to avoid infection, 37% (121/328) of our respondents cited washing hands and good hygiene, 28% (92/328) wearing a mask, 13% avoiding infected others and 12% (39/328) shunning crowded places.

**Table 1:** Behavioural change and risk perceptions in response to the H1N1 pandemic

Variables	Malaysia	Europe	Differences between regions (*x*^2 ^value)	Total
	No (%)	No (%)		N (%)
*Relevant behaviours*				
Public transport use as a result of swine flu				
Less than usual	84 (48)	32 (22)	21.91**	116 (36)
The same or more	93 (53)	112 (78)		204 (64)
Cancellation/delaying of flights				
Yes	99 (56)	25 (17)	49.20**	124 (39)
No	79 (44)	119 (83)		188 (61)
Purchased goods (e.g. face masks)				
Yes	14 (8)	11 (7)	0.14	25 (8)
No	166 (92)	137 (93)		303 (92)
Intend to purchase goods				
Yes	74 (41)	22 (15)	27.03**	96 (29)
No	106 (59)	126 (85)		232 (71)
				
*Risk groups*				
Pig farmers				
More risk than me	157 (88)	72 (49)	68.03**	229 (70)
Same risk as me	16 (9)	74 (50)		90 (28)
Less risk than me	5 (3)	2 (1)		7 (2)
General farmers				
More risk	61 (35)	29 (20)	11.44**	90 (28)
Same risk	108 (61)	116 (78)		224 (69)
Less risk	8 (5)	3 (2)		11 (3)
The elderly				
More risk	87 (49)	96 (65)	8.27*	183 (57)
Same risk	76 (43)	42 (28)		118 (36)
Less risk	13 (7)	10 (7)		23 (7)
Homeless				
More risk	96 (55)	74 (50)	10.23**	170 (53)
Same risk	67 (38)	73 (49)		140 (43)
Less risk	12 (7)	1 (1)		13 (4)
Homosexuals				
More risk	56 (32)	11 (8)	31.82**	67 (21)
Same risk	115 (65)	134 (92)		249 (77)
Less risk	5 (3)	1 (1)		6 (2)
Prostitutes				
More risk	106 (60)	64 (44)	12.10**	170 (53)
Same risk	66 (37)	82 (56)		148 (46)
Less risk	5 (3)	1 (1)		6 (2)
Those already with a disease and with weakened immunity				
More risk	156 (88)	127 (86)	3.49*	283 (87)
Same risk	13 (7)	18 (12)		31 (10)
Less risk	8 (5)	3 (2)		11 (3)
				
*Personal Anxieties*				
Concern about getting flu				
Very concerned	75 (42)	8 (5)	91.67**	83 (26)
Somewhat concerned	52 (29)	30 (20)		82 (25)
Only a little concerned	39 (22)	48 (33)		87 (27)
Not at all concerned	12 (7)	61 (42)		73 (23)
Control over whether infected				
Great deal	35 (20)	19 (13)	2.83	54 (16)
Little	97 (55)	90 (61)		187 (57)
None	46 (26)	38 (26)		84 (26)
Contacted friends to discuss threat				
Yes	33 (19)	90 (63)	66.56**	123 (39)
No	144 (81)	52 (37)		196 (61)
Contacted family to discuss threat				
Yes				
No	49 (28)	31 (22)	1.54	80 (25)
	119 (73)	113 (79)		242 (75)

Note

Asterisks indicate significant regional differences (Europe vs. Asia) using Pearson chi-square statistic * p < .05; ** p < .01. We reran these analyses using logistic regressions controlling for age and sex, with similar results to the chi-square analyses. Further details of these findings are available from the first author.

Percentages are rounded so may not all always add to 100. *N*s range from 312-328 due to some missing data from Malaysia.

### 'Risk groups' and personal anxiety

Five groups were seen as particularly at risk by more than half of our respondents: those with weakened immunity, pig farmers, the elderly, the homeless and prostitutes/highly sexually active. Malaysians were more likely to see pig farmers, general farmers, homosexuals and prostitutes as at greater risk (respective *x*^2 ^(2) = 68.03, 11.44, 31.82, and 12.10, *p *< .001 for each), Europeans were more likely to see the elderly and those with weakened immunity at risk (respective *x*^2 ^(2) = 8.27, 3.49, p < .05). Whilst around half (165/325) of our respondents reported they were at least 'somewhat concerned' about being a victim of the pandemic, this anxiety was stronger in Malaysia, where 71% (127/178) indicated they were at least 'somewhat concerned' (*x*^2 ^(3) = 91.67, *p *< .001). Nearly three quarters of our overall respondents (241/325) felt they had at least some control over whether they were infected. Europeans were more likely to have discussed their fears with their friends (90/142) (*x*^2 ^(1) = 66.56, *p *< .001). Across the sample, personal perceptions of risk about the pandemic were related to those of families and friends (respective *r*s .57, .58, *p *< .001). Those most anxious about personally being a victim of the outbreak were the most likely to reduce their use of public transport (*r *(320) = .48, *p *< .001) and cancel/delay air travel (*r *(322) = .37, *p *< .001).

### Seasonal flu and pork consumption

In our additional (Europe only) data, respondents underrated the dangers of ordinary season flu, with 64% (95/148) claiming that this killed under 100,000 worldwide (actual numbers are between 250,000 and 500,000)[15]. 26% (38/148) of our European respondents wrongly believed that a vaccination for seasonal flu gave immunity against swine flu. The same percentage believed swine flu symptoms differ from those of seasonal flu. While only 3% (4/148) had already obtained anti-viral drugs for use against swine flu, 32% (47/148) claimed they would like to have these at home in case of infection. Few (7%, or 10/148) claimed they had stopped or reduced their eating of pork as a result of the pandemic.

## Discussion

At present, it is unclear as to whether the outbreak of Influenza A, H1N1 will prove to be a "false alarm", or whether the virus will mutate and spread in a new, more dangerous form, perhaps this Autumn [16]. Our data collection in the early stages of the pandemic/pandemic of Spring 2009 suggests that respondents felt they had some control over potential infection. Respondents identified 'washing hands', avoidance of infected people, avoidance of crowded areas and mask wearing as strategies for avoiding infection, reflecting generally approved public health measures [17]. Malaysians were particularly anxious about a pandemic, despite the lack of cases of this influenza in Malaysia during our research period, probably reflecting the recent avian influenza alert in this country [8]. As with previous health alerts, personal anxieties can feed behavioural changes [18], with many Malaysians contemplating significant changes in their use of transport, and anticipating the purchasing of goods, particularly masks, in preparation. European respondents were particularly likely to have discussed the pandemic with their friends, while a quarter of respondents overall had discussed the pandemic thereat with their family. Our correlational data suggests that such conversations may reinforce existing levels of anxiety. Practitioners need to be aware that rumours spread fast during times of pandemic threat, with significant risks of emotional as well as physical 'contagion' between populations [19]. Any increase in anxiety can lead to rapid behavioural changes that can soon lead to shortages, and enhance the desire for medication available at home.

An unrealistically optimistic belief that others are at greater risk than ourselves can reduce our willingness to enact healthy behaviours [20]. During pandemics, particular 'out-groups' may be vulnerable to discrimination [21]. Although respondents correctly identified groups such as the immunocompromised as at greater risk [22], half our respondents saw the sexually active as at greater risk, almost a third of Malaysians suggested homosexuals were at more risk of infection. This may reflect a popular belief in Malaysia that homosexuals are likely to be already immunocompromised through infection with HIV/AIDS. The homeless were also perceived as at greater risk in both Malaysia and Europe. Political and health authorities need to be wary of increased stereotyping and prejudice towards particular societal groups during an influenza pandemic. Our Europe-only data suggested that individuals underestimate the threat of regular seasonal flu, while a quarter of our respondents incorrectly believed seasonal flu and swine flu symptoms were different, and that seasonal flu vaccination could help immunise against swine flu. Despite major media and governmental campaigns across Europe, there is obviously still a need for greater information with respect to symptom logy and immunisation against infection.

Our study was a rapid, cross-sectional analysis in response to a particular outbreak, and as such suffers from a number of limitations. Although this research was unique in tracing initial behavioural responses to this pandemic, our respondents were not true random samples in either continent, and we assessed only a small range of potential behaviours. Our Malaysian sample was drawn from one large city - Kuala Lumpur - and may therefore not be representative of other, more rural populations in that country. Similarly, our European data was drawn primarily from the UK and therefore cannot be seen as representative of the whole continent. Self-report biases in questionnaire completion may mean that our respondents were unwilling to provide openly prejudicial responses, whilst our study in Europe was further limited by including only those who had access to the Internet. To fully model likely behavioural changes, and their consequences for public health services, larger, more representative longitudinal studies are now needed to track public anxieties and health behaviours in the continuing battle against pandemic influenzas.

## Conclusion

Numerous studies have identified behavioural interventions valuable in prevention of epidemic/pandemic influenza, but we know little about individuals' own perceptions of risk at the beginning of a pandemic, which groups in society they believe most at risk of infection and how they have changed and intend to change their behaviours as a pandemic develops. Our findings suggest culture and individual anxiety are important predictors of behavioural responses to pandemic influenza, with higher levels of anxiety about swine flu in Malaysia compared to Europe, and with greater levels of behavioural change in Malaysia. Particular 'out-groups' (e.g. prostitutes, homosexuals) were judged to be at relatively high risk of infection, with Malaysian respondents particularly likely to emphasise the infection risk in these groups. Such judgements of risk may have important implications for the equitable treatment of socially marginalised group, particularly as the pandemic continues to accelerate worldwide.

## Competing interests

The authors declare that they have no competing interests.

## Authors' contributions

RG, SH and LM designed conceived the overall project, and analysed the data along with SH and FN. LM and SH helped in data interpretation and all the authors were involved in final article drafting.

## Pre-publication history

The pre-publication history for this paper can be accessed here:

http://www.biomedcentral.com/1471-2334/9/166/prepub
